# Podocyte Senescence and Aging

**DOI:** 10.34067/KID.0000000000000284

**Published:** 2023-10-31

**Authors:** Stuart J. Shankland, Andrew D. Rule, J. Nathan Kutz, Jeffrey W. Pippin, Oliver Wessely

**Affiliations:** 1 Division of Nephrology, Department of Medicine, University of Washington, Seattle, Washington; 2 Division of Nephrology, Department of Medicine, Mayo Clinic, Rochester, Minnesota; 3 Department of Applied Mathematics, University of Washington, Seattle, Washington; 4 Department of Cardiovascular & Metabolic Sciences, Lerner Research Institute, Cleveland Clinic Foundation, Cleveland, Ohio

**Keywords:** aging, glomerulus, glomerular endothelial cells, mesangial cells, p16/INK4A, parietal epithelial cells, podocytes, senescence, apoptosis, cell activation, cell biology and structure, chronic glomerulonephritis, glomerular disease, glomerular filtration barrier, nephrology, proteinuria

## Abstract

As the population in many industrial countries is aging, the risk, incidence, and prevalence of CKD increases. In the kidney, advancing age results in a progressive decrease in nephron number and an increase in glomerulosclerosis. In this review, we focus on the effect of aging on glomerular podocytes, the post-mitotic epithelial cells critical for the normal integrity and function of the glomerular filtration barrier. The podocytes undergo senescence and transition to a senescence-associated secretory phenotype typified by the production and secretion of inflammatory cytokines that can influence neighboring glomerular cells by paracrine signaling. In addition to senescence, the aging podocyte phenotype is characterized by ultrastructural and functional changes; hypertrophy; cellular, oxidative, and endoplasmic reticulum stress; reduced autophagy; and increased expression of aging genes. This results in a reduced podocyte health span and a shortened life span. Importantly, these changes in the pathways/processes characteristic of healthy podocyte aging are also often similar to pathways in the disease-induced injured podocyte. Finally, the better understanding of podocyte aging and senescence opens therapeutic options to slow the rate of podocyte aging and promote kidney health.

## Introduction

**Figure 1:**
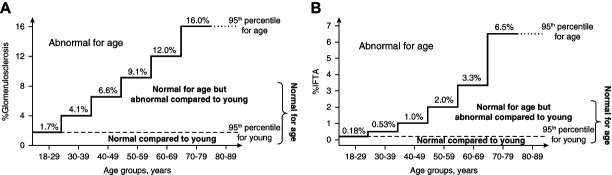
**Thresholds in normotensive living donor kidneys at donation.** (A) Percentage of glomerulosclerosis on the basis of the number of globally sclerotic glomeruli divided by all glomeruli detected in the biopsy. (B) Percentage of interstitial fibrosis and tubular atrophy (IFTA) on the basis of morphometric annotations of IFTA and cortex in the biopsy.^6^ Graphs are based on the 95th percentile for each age group; the line for young (18–29 years) is expanded for reference.

As life expectancy increases, the effect of advanced age on kidney health and function is becoming an increasingly important medical and socioeconomic factor. GFR declines after age 40 years by 0.8%–1.0% per year,^1,2^ and kidneys from healthy kidney donors aged 70–79 years have 400,000 fewer intact nephrons compared with young kidney donors aged 18–39 years.^3,4^ Age-dependent glomerulosclerosis in humans occurs predominately in the superficial cortex and seem to associate with glomeruli that have an ischemic appearance (tuft deflation, capsule thickening, and periglomerular fibrosis).^5^ Among healthy normotensive kidney donors, there is a marked increase from ages 18–29 years to 70 years and older in the 95th percentile for percentage of glomeruli that are globally sclerosed (1.7%–16%) and the percentage of cortex that has interstitial fibrosis and tubular atrophy (0.18%–6.5%) (Figure 1).^6,7^ This aging process involves changes in number, structure, and function to all four resident glomerular cell types—the podocyte, endothelial, mesangial, and parietal epithelial cells.^8–13^ Floege *et al.*^14^ were the first to describe age-related glomerulosclerosis as a podocyte disorder, an observation subsequently confirmed by others.^12,15^ This review will highlight published reports on podocyte senescence and changes to podocytes with progressive aging.

## Senescence and Aging

Aging is defined as the process of growing old, whereas senescence is the process of cell deterioration that occurs with aging (Table 1).^16^ Senescence comes in different forms and is a highly dynamic and regulated state typified by telomere shortening, increased lysosomal activity, macromolecule accumulation, and cell cycle arrest in dividing cells. The latter is referred to replicative senescence (Table 1).^16^ Senescent cells accumulate with increasing chronological age, but can also be triggered by a variety of stressors, such as DNA damage, nutrient deprivation, hypoxia, and mitochondrial dysfunction. Senescence is believed to prevent damaged cells from proliferating, and initial studies focused on dividing cells. However, since then, it has become evident that post-mitotic terminally differentiated cells similarly undergo cellular senescence.^17^ This includes podocytes that undergo cell cycle arrest on differentiation.^18–22^ Thus, at least in post-mitotic cells, the pathways required for cell cycle arrest and those for senescence are independent.^17^ Finally, it is important to emphasize that senescence is a double-edged sword. By acquiring a senescence-associated secreted phenotype (SASP) and releasing cytokines and growth factors, senescence supports cellular regeneration and repair over a short time window. Yet, chronic exposure to SASP can induce senescence and injury in neighboring cells ultimately causing tissue damage.^23–25^ The clinical relevance of the accumulation of senescent kidney cells in aged mice is illustrated by the INK-ATTAC mouse model, where senescent p16^INK4^-positive cells were specifically ablated.^26^ These mice exhibit reduced age-associated glomerulosclerosis and reduced tissue damage of the surrounding cells.

**Table 1:** Senescence and aging definitions

Process	Type	Definition
Senescence	Stress-induced premature senescence	The process of cell deterioration due to stressors, such as injury
	Replicative senescence	The process of cell deterioration with age
	Premature senescence	Cell deterioration preceding that seen in healthy aging
	Paracrine senescence	Cell deterioration induced by neighboring cells through secretion of SASPs
	Post-mitotic cellular senescence	A post-mitotic (nondividing) cell that entered senescence in response to stress or during aging
Aging	Aging	The process of growing old
	Healthy aging	Growing old with no clinical signs of disease or infection
	Premature aging	An aged phenotype preceding that is seen in healthy aging
	Aged phenotype	Reduced life span and health span

SASP, senescence-associated secreted phenotype.

## Podocyte Senescence

In podocytes, senescence increases with healthy aging, but also can undergo stress-induced senescence during disease.^18–21^ Because podocytes are terminally differentiated epithelial cells, they have exited the cell cycle and reside in G_0_; thus, the criteria qualifying them as senescent are different from proliferating cells. Table 2 summarizes and contrasts these characteristics by comparing non-senescent and senescent podocytes as well as senescent, proliferating epithelial cells. Markers of senescence expressed in podocytes in human and mouse kidneys with advancing age include senescence-associated *β*-galactosidase staining and lipofuscin staining and an increase in p16, p19, p21, and p53 mRNA and protein levels.^9,27–32^ Similarly, proteasome impairment results in an increase of p19 levels.^33^ It is, however, important to note that molecular signatures for aging and senescence are still only partially established for any cell type or organ. Therefore, there are ongoing efforts to deepen our understanding, such as the Common Fund's Cellular Senescence Network (SenNet).

**Table 2:** Comparison of non-senescent and senescent podocytes and proliferating epithelial cells

Characteristics	Non-Senescent Podocytes	Senescent Podocytes	Senescence- Proliferating Epithelial Cells
Cell cycle phase	G_0_ cell cycle arrest	G_0_ cell cycle arrest	G_1_ and occasionally G_2_ cell cycle arrest
Cell cycle re-entry	No	No	Yes, when stress is reduced
SA-*ß*Gal staining	Negative	Positive	Positive
Lipofuscin staining	Negative	Positive	Positive
Telomere length	Assumed normal	?	Shortened (in human cells)
SASP	Not detected	Yes	Yes
Morphologic changes	Not detected	Widened slit diaphragms	Flattened appearance
p16/INK4A	Not detected	↑	↑
p19/INK4D	Not detected	↑	?
p21/CIP1	Not detected	↑	↑
p27/KIP1	↑	↑	↑
p57/KIP2	↑	↓	Not detected
p53	Not detected	↑	↑
Apoptosis	Prone	Resistant	Resistant
Polyploidy	Not detected	Yes	Yes
Hypertrophy	Not detected	Yes	Yes

↑, increased; ↓, decreased; ?, unknown; SA-*ß*-Gal, senescence-associated *β*-galactosidase staining; SASP, senescence-associated secreted phenotype.

## Podocyte Aging Phenotype

**Figure 2:**
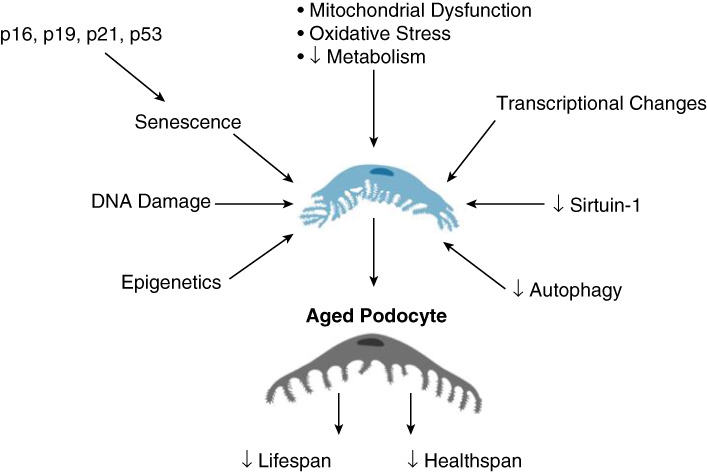
**Schematic of pathways reducing podocyte life and health span*.*** See text for details. Created in part with Biorender.com.

In addition to senescence, the aged podocyte phenotype is characterized by several hallmarks, including foot process and ultrastructural changes; hypertrophy; endoplasmic reticulum, cellular, and oxidative stress; and reduced autophagy (Table 3). One compelling interpretation of podocyte aging is the concept of life and health span (Table 3). The life span of an individual podocyte corresponds to how long a podocyte is alive and as such is definitive. The health span, on the other hand, is the part of an individual podocyte's life spent in good health and able to perform the cellular and molecular functions to maintain its physiology, structure, and function. In contrast to the life span, health span is not abrupt, but is best considered as continuous, changing dynamically throughout life. There are many molecular pathways that are associated with the podocyte health span. Many are intrinsic to podocytes and seem to change with increasing age (Figure 2). Others are extrinsic confounders and include known podocyte stressors, such as obesity, hypertension, premature birth, and glomerular disease.^34^

**Table 3:** Characteristics of the podocyte aging phenotype

Process	End point	Measurement
Aging Classical hallmarks observed in podocytes	↓Foot process length and ↑slit diaphragm width	Podocyte exact morphology measurement procedure and filtration slit density
	Hypertrophy	↑Podocyte nuclear area/podocyte volume
	↓Ultrastructure	Expansion microscopy, actin cytoskeleton
	↓Autophagy	mTOR, ATG/MAP1LC3, p62
	↓Ubiquitin-proteosome	↑p19ARF
	Cell stress	↑Desmin
	Endoplasmic reticulum stress	↑GRP94/Hsp90b1
	Oxidative stress	↑Reactive oxygen species, Nox 4, Ncf1, Ncf2, Prkcd ↓Ndufa5, Ndufs2,4, Sdha, Cox5b,6c,7b
	↑Aging genes	↑*Gpnmb, Ighm, Igkc, J Chain, Ms4a7, Rplp2, Ccr1, Emp3, Fn1, Vcam1, Selplg*
	↑Senescence-associated secretory phenotype (SASPs)	↑*Il1a, Mmp12, Ccl5, Osm, Areg, Il1b, Mmp13, Hgf, Cxcl3, Il6, Mmp3, Ccl8, Il13, Csf2, Icam1, Mmp14, Cxcl1, Tnsf11b*
Life span Length of the time a podocyte lives; corresponding to the total number of podocytes comprising the glomerular filtration barrier	↓Podocyte number/density	↓p57 and ↓p57/glomerular size
	↑Apoptosis	↑Cleaved caspase-3, TUNEL
	↑Pyroptosis	↑Caspace-1, NLRP3, Gasdermin D
	↑Detachment	Podocyturia
	Cell cycle arrest	BrdU, DNA content by Flow cytometry
	Polyploidy	Flow cytometry, PAS staining
	Mitotic catastrophe	Flow cytometry, PAS staining
Health span Part of a podocyte's life that is spent in good health to perform normal cellular and molecular functions that maintain physiology, structure, and function	↓Canonical genes/proteins	↓*Actn4, Cdkn1, Col4a, Fat1, Lamb2, Nphs1, Nphs2, Podxl, Synpo*
	↓Canonical transcription factors	↓WT1, LMX1B, FOXC2, MafB
	↓Function	↓VEGF, angiopoietin-1
	↑Inflammation	↑PD1, PD-L1, PD-L2, *Naip6, Nlrp3, Aim2, Naip5, Nlrp1b, Nlrc4, Naip2, Casp1, Gsdmd, Casp4, Nlrp1a, Pycard, TLR 8,3,4,2*

↑, increased; ↓, decreased. VEGF, vascular endothelial growth factor.

## Reduced Podocyte Life Span with Aging

With advancing age, the absolute and relative numbers of podocytes decrease.^9^ A human glomerulus contains 589±166 podocytes according to Wiggins *et al.* or 558 podocytes according to Puelles *et al.*^35,36^ During aging, podocyte numbers drop by approximately 0.9% per year from more than 300 podocytes per 10^6^ *µ*m^3^ in young people to <100 per 10^6^ *µ*m^3^ in 70–80-year-old people.^15^ Stereologic calculations suggest that a human loses 5.6 million podocytes per kidney per year and that most podocyte depletion occurs by glomerulosclerosis-associated glomerular loss rather than loss of individual podocytes in nonsclerotic glomeruli.^37^ This is also seen in aged rats^38^ and mice.^9,39–43^ Compared with young mice, podocyte density is 46% and 39% lower in the cortex and juxtamedulla of aged mice, respectively.^39^ However, how this relates to the other glomerular cell types is still inconsistent and warrants further investigation.^18,44–47^

Animal model studies have shown a direct correlation between podocyte depletion and glomerulosclerosis.^48–50^ When glomerulosclerosis increases with age,^7,51–53^ this is paralleled by a decrease in absolute podocyte numbers and density.^9,38–44,54^ This age-dependent podocyte depletion has been shown to be clinically highly relevant. A decrease in absolute podocyte number and density eventually results in glomerulosclerosis,^45,55^ and thus, podocyte loss is an important predictor for glomerulosclerosis risk in healthy aged kidneys.^15,36,56,57^ Human kidney transplants with low podocyte endowment develop transplant glomerulopathy at a higher rate compared with those with normal podocyte density.^58,59^ Although it is unclear which is the predominant cause of reduced podocyte life span, apoptosis,^60,61^ pryoptosis,^62^ detachment,^15^ mitotic catastrophe, and DNA damage^19^ are all mechanisms increased in aged podocytes. Recent studies have shed light on epigenetic influences on podocyte aging. A loss of the chromatin modifiers HDAC1 and HDAC2 in podocytes results in sustained DNA damage and podocyte senescence.^22^ Similarly, aging (including a decrease in podocyte density) was phenocopied in the inducible changes to the epigenome mouse strain, which introduces double-stranded DNA breaks resulting in a loss of epigenetic information.^63^ Finally, there are progerias, syndromes that exhibit features of premature aging due to mutations in genes responsible for DNA repair, replication, and transcription.^64^ Although still understudied with respect to podocytes, one progeroid mouse model describes a severe reduction in podocyte foot processes.^65^

Regardless of the cause, terminally differentiated podocytes are post-mitotic and thus are unable to self-renew and replace those podocytes lost due to aging.^66^ Thus, to retain podocytes covering the glomerular basement membrane during aging, podocytes rely on compensatory hypertrophy.^44^ The average volume of a human podocyte is 335±136 um^3^ in healthy humans.^35^ Yet, podocyte cell volume and nuclear size increase annually by 3.2% and 2%, respectively.^15^ While initially beneficial, this compensatory hypertrophy eventually becomes maladaptive resulting in glomerulosclerosis.^12,15,44^ Finally, during aging with usual comorbidity, the decrease in podocyte number accompanied by an increase in overall glomerular size leads to a decrease in podocyte density.^15,66^ However, one cannot simply associate these changes with aging only. There are parallel adaptive processes to maintain glomerular physiology with age-related comorbidities. In the absence of common comorbidities, such as obesity, diabetes, and hypertension, single nephron GFR, glomerular volume, and glomerular filtration capacity do not seem to change with age in healthy humans.^67–69^

## Changes to Podocyte Health Span with Aging

**Figure 3:**
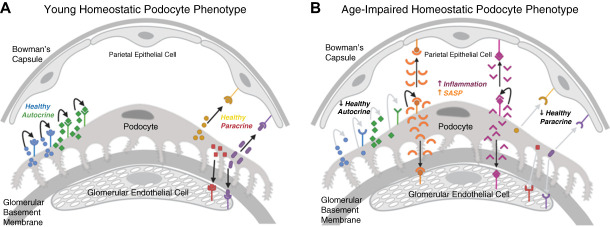
**Signaling in young and old glomeruli*.*** (A) Schematic of the autocrine and paracrine loops occurring in the glomerulus of a young kidney and (B) how they are altered in an aged kidney. Autocrine and paracrine loops between podocytes and parietal epithelial cells and glomerular endothelial cells are indicated; for simplicity, mesangial cells have been omitted (but will likely show similar cross-talk). SASP, senescence-associated secreted phenotype. Created with Biorender.com.

These phenotypic changes are paralleled by molecular changes (Table 3). They include a decrease in the expression of canonical podocyte genes and proteins, changes in the actin cytoskeleton, mitochondrial and metabolic dysfunction, and reduced autophagy.^34^ One intriguing, yet not well-described aspect is a reduced synthetic function best exemplified by a meta-analysis of recent RNAseq data.^19^ In this study, we identified 55 ligand-receptor interactions that were specifically enriched in healthy podocytes. While the individual contributions of these to podocyte health need to be evaluated, it is noteworthy that 26 of these receptor-ligand pairs were significantly reduced in aged podocytes. These data suggest that aging podocytes are characterized by the lack of or reduced activity of autocrine and perhaps paracrine signaling networks, such as vascular endothelial growth factor (Figure 3).^19^ This may explain why glomerular endothelial cell numbers are reduced in aged rats and mice.^18,70^

## The Aging Inflammatory Phenotype

In addition to aging leading to the deterioration of cellular functions, there is an increasing body of evidence showing that aging podocytes acquire an inflammatory phenotype. However, unlike systemic inflammation (which also occurs in aging), this is a sterile form of intracellular inflammation. It does not involve inflammatory cells but involves changes in the local microenvironment. Although it has not yet been mechanistically proven for the glomerulus, it is tempting to speculate that—on the basis of analogy to other organ systems^17^—the soluble proinflammatory factors released by podocytes have deleterious paracrine effects on neighboring glomerular cells, that is, parietal epithelial cells, mesangial cells, endothelial cells, and even neighboring podocytes themselves (Figure 3). Among these, the following are the best understood in causing the aging podocyte inflammatory phenotype:

- Senescence-associated secretory phenotype: Although senescent cells are in a growth arrested state, they remain metabolically active and exhibit a hypersecretory phenotype called the SASP. It constitutes a major hallmark of senescent cells in general and is responsible for many of their pathophysiologic effects. Yet, it is important to note that the regulators of senescence and that of SASP differ. The main components of the SASP are soluble signaling factors, such as proinflammatory cytokines and growth factors, proteases, extracellular matrix proteins, and matrix metalloproteinases. Current work has demonstrated that several of these are also detected in aged mouse podocytes (Table 2).^19^ While likely still an incomplete list, the SASP may trigger paracrine senescence in nearby cells in other organs.^17^
- NLRP3 inflammasome: The NLRP3 inflammasome is a multiprotein complex involved in the innate immune response and inflammatory signaling.^71,72^ It is typically activated by pattern- and damage-associated molecular patterns.^71,72^ NLRP3, a major constituent of the inflammasome, and its downstream effector Caspase-1 are increased in podocytes from aged mice.^19^ Active Caspase-1 causes cell death by pyroptosis and the generation of proinflammatory interleukins-1*ß* and -18, which are increased in aged mouse podocytes as early as middle-aged. In humans, NLRP3 was not detected in glomeruli from young healthy people but was increased in glomeruli of older people. Moreover, in microdissected human glomeruli, increased *NLRP3* mRNA expression was associated with an increased percentage of globally sclerosed glomeruli, higher glomerular volume, and reduced podocyte density.^62^ More importantly, the NLRP3 inflammasome plays a similarly important role in podocyte aging as it does for glomerular disease under non-aged conditions.^73–81^ Inhibiting its activity in middle-aged mice with the NLRP3 inhibitor MCC950 and deleting NLRP3 genetically slowed aging, as evidenced by a higher podocyte density and higher levels of health span markers, reduced podocyte senescence, and SASP.^62^
- Protein cell death protein-1 (PD-1): The PD-1 pathway is best known in the cancer field for its role in the induction and maintenance of immune tolerance within the tumor microenvironment by inhibiting T-cell activation, proliferation, and survival.^82^ There is a significant increase in the expression of PD-1/PDCD1 and its two ligands PD-L1 and PD-L2 in aged podocytes compared with young podocytes.^18^ In humans, glomerular *PCDC1* transcripts increase progressively with aging and this increase correlates with lower eGFR, higher segmental glomerulosclerosis, and vascular damage. In kidneys of aged mice, blocking PD-1 with an antibody improves the aging phenotype in both life and health span. Moreover, ectopic expression of PD-1 in cultured podocytes triggers a partially Caspase-3–dependent apoptosis.
- Other forms of inflammation: Gene set enrichment analysis of aging mRNAseq data from aging mice implicates additional inflammatory pathways, such as IL2, IL6, INF*γ*, TNF, and complement signaling.^62^ Yet, whether and how they affect glomerular aging still needs to be assessed.

## Mitigating Podocyte Aging

As with aging in general, there is the question whether mitigation of podocyte or even kidney aging should be a clinical goal. This is an ongoing discussion. However, in our opinion, it should be given consideration under certain circumstances when an aged kidney is placed under added stress that might further accelerate aging and disease progression (*e.g.*, FSGS in the elderly, reduction in nephron mass due to cancer resection, transplantation of older kidneys, obesity, and hypertension). The following approaches/treatments have been reported to slow and even reverse podocyte aging:

- Calorie restriction: Wiggins *et al.*^44^ showed that compared with an *ad libidum* diet, calorie-restricting rats prevented age-associated podocyte hypertrophy, improved the levels of several key podocyte genes, and reduced podocyte stress.^38,44^ While the precise mechanism(s) underlying this effect are still unknown, one candidate pathway is Sirtuin signaling, which is known for being involved in metabolic regulation.^83–85^
- Mitochondrial stabilizers: The mitochondrial antioxidant elamipretide (SS-31) is a small cell-permeable synthetic peptide that improves mitochondrial function.^86,87^ Treatment of old mice with elamipretide preserved podocyte mitochondrial integrity, lowered the expression of the reactive oxygen species-generating enzyme *Nox4*, and increased the electron transfer genes *Ndufa9* and *Cox4i1.*^9^ At the cellular level, elamipretide reduced podocyte hypertrophy, foot process effacement, and injury, while improving podocyte density.^9^ Yet, the most unexpected finding of the study was that despite the advanced kidney age of 24 months, elamipretide still preserved the remaining mitochondrial integrity and significantly improved podocyte health.
- Senolytics: Small-molecule senolytic drugs that selectively clear senescent cells have been used with some success in experimental nonglomerular kidney diseases.^88–91^ US Food and Drug Administration–approved senolytics for non-kidney diseases include the pan-tyrosine kinase inhibitor dasatinib and the flavonoids, quercetin and fisetin, that interfere with a subset of cellular kinases. Quercetin and dasatinib reduce fibrosis and tubular injury in experimental AKI and aging. Trichostatin A is a small-molecule class I/II histone deacetylase inhibitor that is also a senolytic. It is used to treat cutaneous T-cell lymphoma.^92^ Yet, it may have the opposite effect in the glomerulus because inhibiting/deleting HDAC1/2 in podocytes enhances rather than improves senescence.^22^ Finally, it is important to note that removing senescent podocytes from glomeruli may not be the most advisable therapeutic strategy because removal of senescent podocytes causes podocyte depletion, which equates to glomerular injury.
- Senomorphics: This class of small molecules aims to suppress all or at least several characteristics of the senescence phenotype without killing the cells.^93^ Among those, metformin and rapamycin inhibit senescence and SASP by inducing autophagy, thereby reducing the accumulation of damaged organelles. Similarly, Janus kinase inhibitors, such as the US Food and Drug Administration–approved ruxolitinib, tofacitinib, and baricitinib, reduce SASP cytokines.
- Rapamycin: In contrast to other organs,^94^ rapamycin has not shown the same promise in the glomerulus. Rapamycin treatment had no effect on podocyte density in aged mice; it did, however, improve several aspects of parietal epithelial cell aging.^42^
- Checkpoint kinase inhibitors: As discussed above, the PD-1 signaling pathway is increased in podocytes in aged human and mouse kidneys.^18^ Blocking PD-1 reduced podocyte senescence and improved podocyte life and health span in middle-aged mice.^18^ However, because checkpoint kinase inhibitors can cause AKI,^95^ their use is not advocated for in the aged kidney. Further studies are needed to better understand this pathway as a mechanism of podocyte aging.
- NLRP3 inflammasome inhibitors: Pharmacologically inhibiting the increase in NLRP3 in middle-aged podocytes with MCC950 reduced their senescence and improved many measures of podocyte life span and health span.^62^

## Outlook

**Figure 4:**
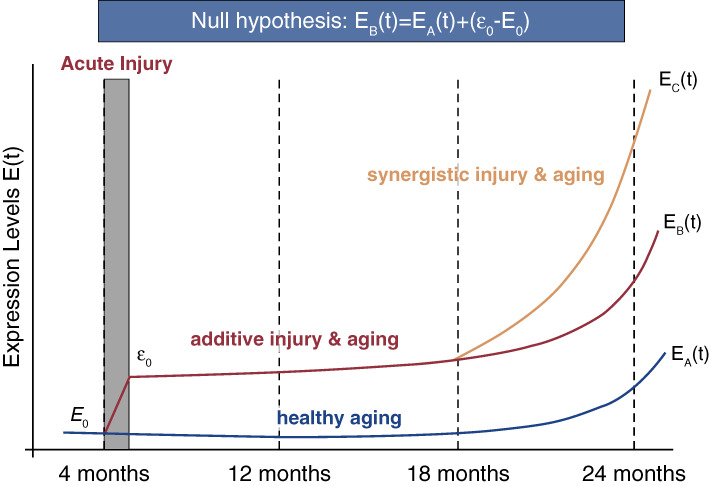
**Mathematical modeling of the interaction between injury and aging*.*** Predictive modeling of changes in expression levels of genes increased by podocyte injury. Expression is depicted as a function of time comparing the 4m injury scenarios with the uninjured situation. The growth model assumes that the rate of change is proportional to the expression level, thus leading to compounding effects and standard exponential growth. The blue line corresponds to the changes in gene expression upon healthy aging. Injury induces a jump in gene expression levels (ε_0_-E_0_). An additive interaction between injury in age is indicated by the red line while a synergistic interaction is depicted by the orange line. A testable null hypothesis that the interaction between injury and age is additive is shown in the blue box.

Glomerular changes with aging are associated with both adaptive and maladaptive features. While maladaptive features that lead to increased susceptibility for CKD in the elderly are well documented, the adaptive features are more difficult to capture. The age-related nephron loss is indolent and progressive. Remnants of incapacitated nephrons occur in the form of glomerulosclerosis, interstitial fibrosis, and tubular atrophy, but are also underdetected because of progressive atrophy and reabsorption of remnant nephrons.^3,4,96^ Thus, new approaches/measurements are needed to provide a sliding baseline of these adaptive processes and integrate them with the maladaptive events. Similarly, many cellular and molecular questions remain unanswered. The precise triggers, the temporal progression of senescence/aging, and the rate of aging of the individual kidney cell types during healthy aging are still largely unknown. In fact, answers may come from unexpected angles. For example, it has recently been shown that in non-kidney organ systems, the reactivation of endogenous retroviruses can trigger/enforce senescence.^97^ However, molecular advances, such as single-cell/nuclear RNA sequencing and spatial transcriptomic approaches, provide unparalleled opportunities. These will allow us to address some of the key questions that are clinically relevant, such as (*1*) how aging podocytes cause paracrine injury to the neighboring glomerular cells, (*2*) under what clinical scenarios slowing down or even reversing of podocyte aging may be therapeutically advantageous, and (*3*) how disease-induced podocyte injury and healthy aging intersect. The latter is particularly germane because epidemiologic data show that the outcomes are worse in glomerular diseases in the elderly, that CKD is more common in the elderly, and that glomerular diseases progress with patient age. This raises the important question whether there is a mechanistic link or intersection between podocyte injury and aging. Future studies need to address whether there is a qualitative and quantitative or even synergistic overlap in the pathways changed in individuals with glomerular disease and those changed with the glomerulosclerosis seen in healthy aging. For example, does disease-induced injury to non-aged podocytes cause premature aging or whether they simply have overlapping, but functionally distinct signatures (see Figure 4 for an experimentally testable theoretical concept). Further studies are needed to precisely interrogate these scenarios and determine whether injury causes a premature senescent phenotype in non-aged podocytes and how this affects the progression of glomerular diseases.
